# Supplementary material for: Toxicity of Cyanopeptides from Two Microcystis Strains on Larval Development of Astyanax altiparanae
Source: Toxins (Basel). 2019 Apr 13;11(4):220. doi: 10.3390/toxins11040220 (PMC6520764; doi:10.3390/toxins11040220)
Supplement: Supplementary file 1 [file toxins-11-00220-s001.pdf]

Supplementary materials: Toxicity of Cyanopeptides from Two *Microcystis* Strains on Larval Development of *Astyanax altiparanae*

Kelly Fernandes, Andreia Gomes, Leonardo Calado, , George Yasui, Diego Assis, Theodore Henry, Ana Fonseca and Ernani Pinto

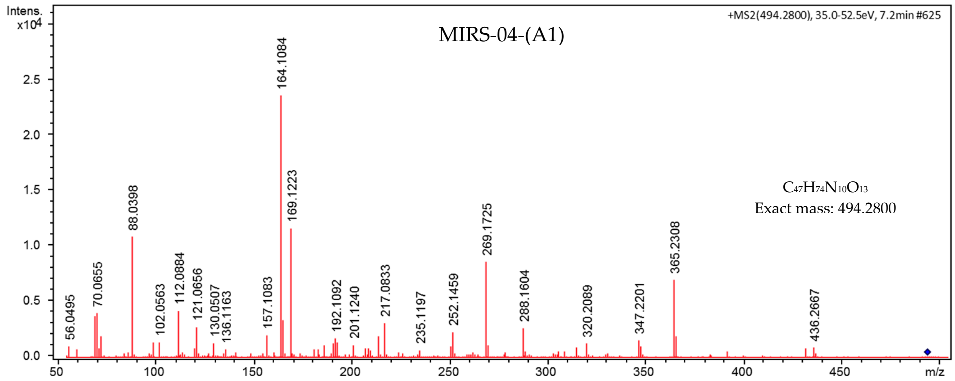

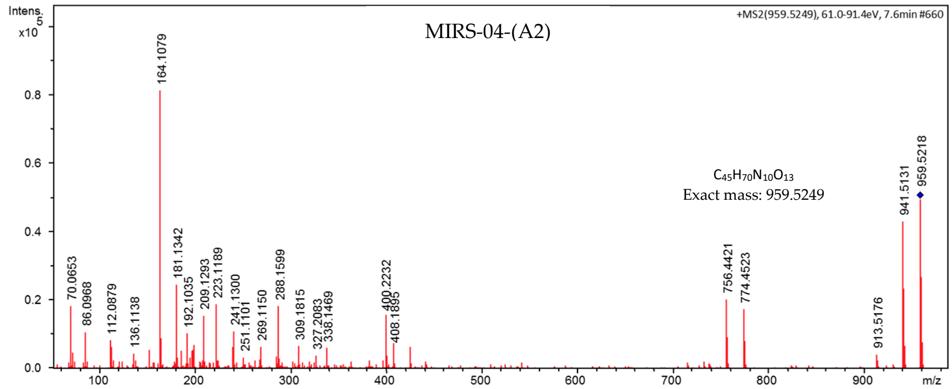

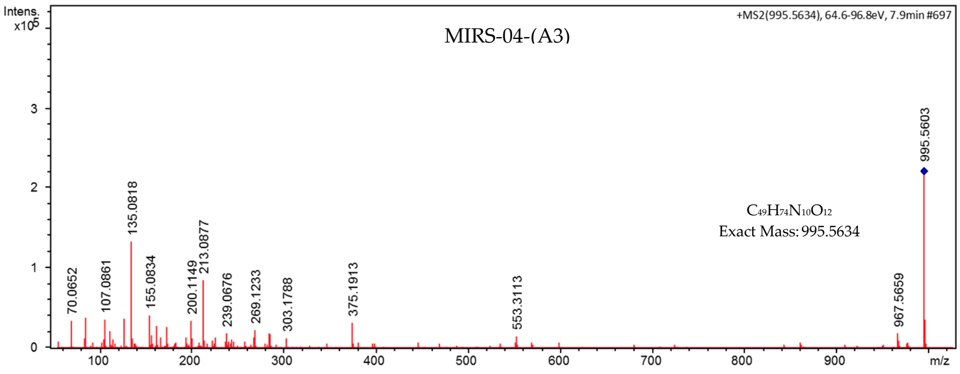

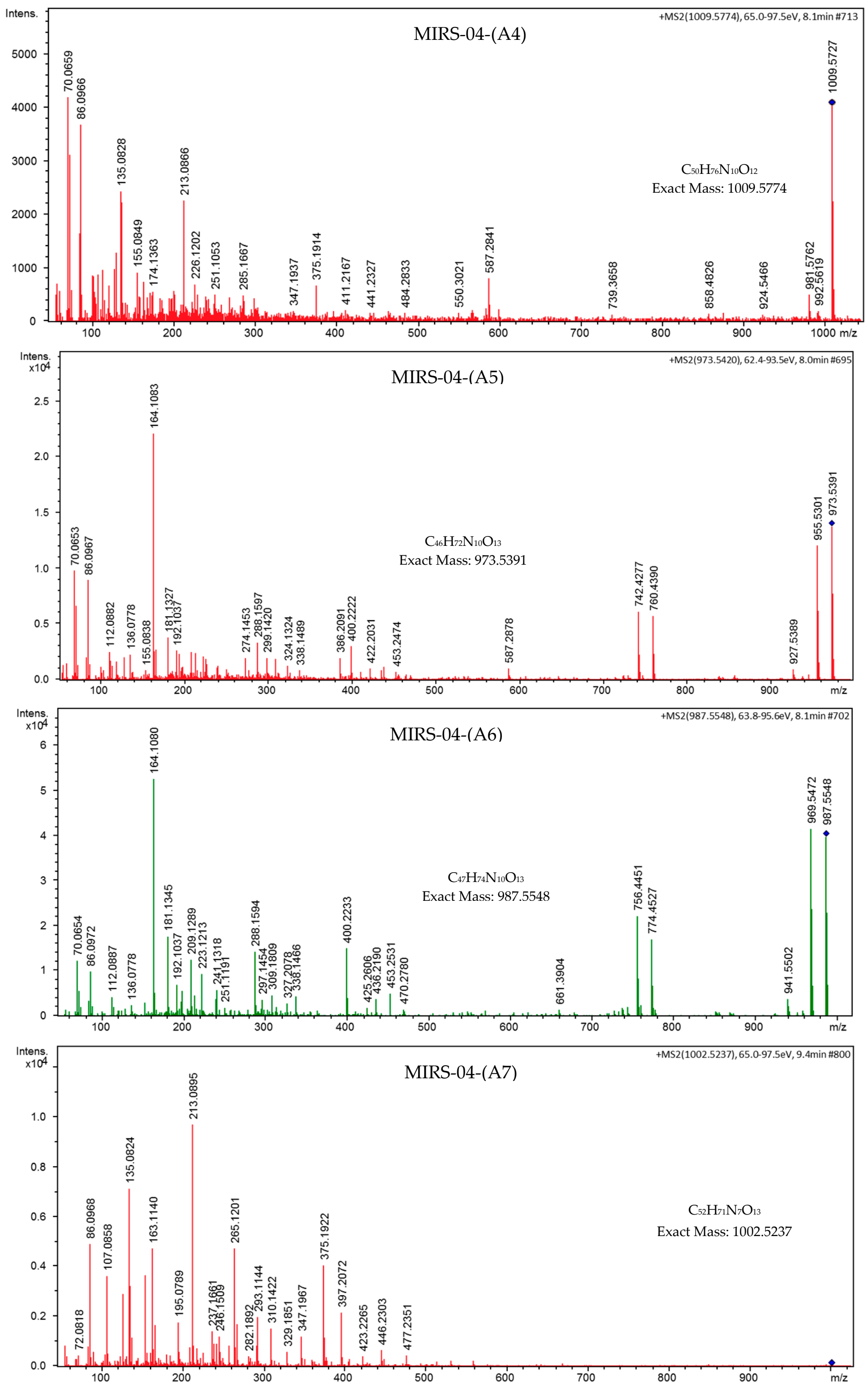

Figure S1. MS[2] spectra ions products of the compounds identified in strain MIRS-04 extract. The mass spectra follow the sequence of the peaks identified in the chromatogram for the extract of strain MIRS-04. The mass spectra are presented and quoted in Figure 1 in the text. Peak A1= $[M+2H]^{2+}$ *m/z* 494.2800 (micropeptin K139), Peak A2= $[M+H]^{+}$ *m/z* 959.5249 (micropeptin 959), Peak A3= $[M+H]^{+}$ *m/z* 995.5634,

Peak A4= $[M+H]^+$ *m/z*1009.5774 (microcystin HilR), Peak A5= $[M+H]^+$ *m/z* 973.5391 (micropeptin 973), Peak A6 = $[M+H]^+$ *m/z* 987.5548 (micropeptin B) and Peak A7= $[M+H]^+$ *m/z* 1002.5237 (microcystin-LY).

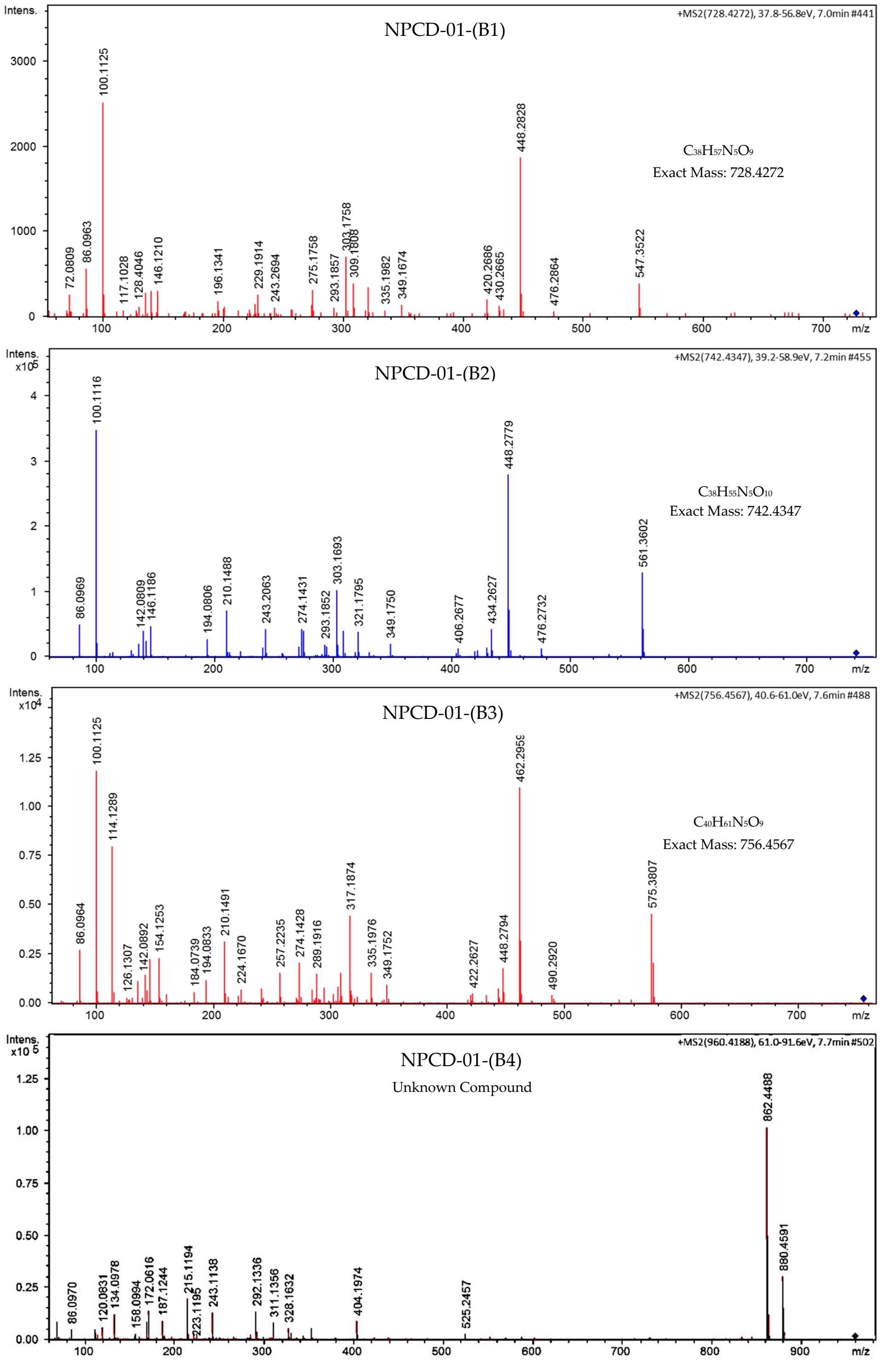

Figure S2. MS² spectra ions products of the compounds identified for the extract of strain NPCD-01. The mass spectra follow the sequence of the peaks identified in the chromatogram for the strain NPCD-01.The mass spectra are quoted as shown in Figure 1 in the text: Peak B1 = $[M+H]^+$ *m/z* 728.4272 (microginin FR1), Peak B2 = $[M+H]^+$ *m/z* 742.4347 (microginin FR2/FR4), Peak B3 = $[M+H]^+$ *m/z* 756.4567 (microginin SD 755) and Peak B4 = $[M+H]^+$ *m/z* 960.4188 unknown compound.

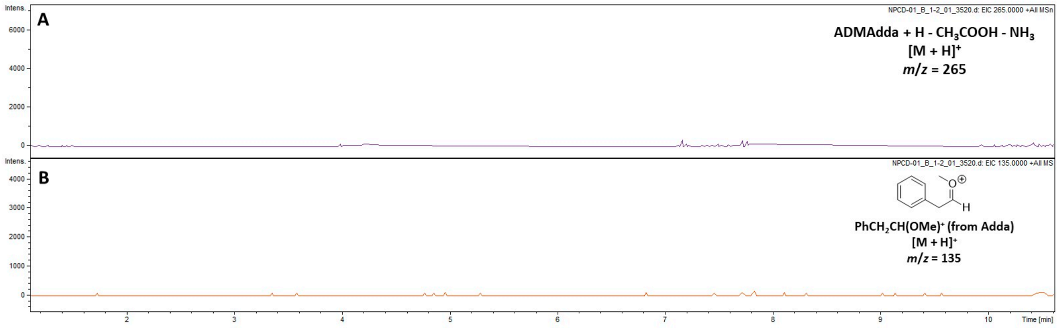

Figure S3. QTOF ESI+ Extracted ion chromatograms (EIC) of (A) ADMAdda + H - CH_3COOH - NH_3 (*m/z* = 265) and (B) $PhCH_2CH(OMe)^+$ (*m/z* = 135) (from Adda), confirming the absence of MCs in the water-soluble extract of NPCD-01 strain.
